# Intra-articular injection of placental mesenchymal stromal cells ameliorates pain and cartilage anabolism/catabolism in knee osteoarthritis

**DOI:** 10.3389/fphar.2022.983850

**Published:** 2022-11-29

**Authors:** Mengqiang Fan, Jingwen Zhang, Li Zhou, Zuxiang Chen, Ronghua Bao, Longpo Zheng, Peijian Tong, Yuhai Ma, Letian Shan

**Affiliations:** ^1^ The First Affiliated Hospital, Zhejiang Chinese Medical University, Hangzhou, China; ^2^ Cell Resource Bank and Integrated Cell Preparation Center of Xiaoshan District, Hangzhou Regional Cell Preparation Center (Shangyu Biotechnology Co Ltd), Hangzhou, China; ^3^ Fuyang Orthopaedics and Traumatology Affiliated Hospital, Zhejiang Chinese Medical University, Hangzhou, China; ^4^ Department of Orthopedics, Shanghai Tenth People’s Hospital, Tongji University School of Medicine, Shanghai, China; ^5^ The Department of Orthopedics, Hangzhou Hospital of Zhejiang Provincial Armed Police Corps, Hangzhou, China

**Keywords:** osteoarthritis, paracrine, conditioned medium, rat, placenta-derived mesenchymal stromal cells

## Abstract

**Background:** Knee Osteoarthritis (kOA), the most common joint degenerative disorder, lacks effective therapeutics. Placenta-derived mesenchymal stromal cells (PMSCs) are effective in tissue repairing and generation, which have potential in treating kOA. This study aimed to determine the anti-kOA efficacy of PMSCs and to explore its action mode.

**Methods:** Flow cytometry and three-line differentiation were performed for identification of PMSCs. *In vivo*, a rat kOA model established by anterior cruciate ligament transection (ACLT) surgery was used to evaluate the efficacy of PMSCs. Histopathological HE and SO staining with Osteoarthritis Research Society International scoring were conducted, and cartilage expressions of MMP13 and Col2 were measured by immunohistochemistry. Pain behavior parameters by mechanical withdrawal threshold (MWT) and thermal withdrawal latency (TWL), were measured. *In vitro*, wound healing and cell immunofluorescence assays were conducted to detect the proliferation and migration ability of chondrocytes treated with PMSCs conditioned medium (PMSCs-CM). Quantitative real-time PCR (qRT-PCR) and Western blot (WB) assays were applied to explore the molecular action of PMSCs on chondrocytes.

**Results:** The results of flow cytometry indicated that the surface markers of PMSCs (CD73 > 95%, CD90 > 95%, and CD34 < 2%) were consistent with the typical mesenchymal stromal cells. The *in vivo* data showed that PMSCs significantly reversed the kOA progression by protection of cartilage, regulation of anabolic (Col2) and catabolic (MMP13) expressions, and relief of pain symptoms. The *in vitro* data showed that PMSCs promoted chondrocyte proliferation and migration and significantly restored the IL-1β-induced abnormal gene expressions of Col2, Mmp13, Adamts4, Adamts5 and Sox9 and also restored the abnormal protein expressions of Col2, Mmp13 and Sox9 of chondrocytes. The molecular actions of PMSCs on chondrocytes in nested co-culture way or in conditioned medium way were similar, confirming a paracrine-based mode of action.

**Conclusion:** This study demonstrated PMSCs’ anti-kOA efficacy and its paracrine-based action mode, providing novel knowledge of PMSCs and suggesting it as a promising cell therapy for treatment of kOA.

## Introduction

Knee osteoarthritis (kOA) is characterized by impaired normal synthesis and degradation of articular cartilage, extracellular matrix and subchondral bone, accompanying with synovial inflammation and synovial fibrosis (Belluzzi et al., 2020; Zhang L. et al., 2021). It is one of the main causes of mobility impairment and chronic pain, affecting almost half of the elderly people worldwide (Musumeci et al., 2015). Several treatment options are available for kOA, including oral nonsteroidal anti-inflammatory drugs, physical therapy, and finally operative treatment (Huang et al., 2015; Szwedowski et al., 2021). However, none of these can effectively delay or reverse the kOA progression or have long-term improvement (Cross et al., 2020). Thus, most patients have to undergo surgery of knee arthroplasty, bringing heavy social and economic burden (Doyle et al., 2020). In China, the number of total knee arthroplasty cases attained 249,000 in 2018, and the total cost reached about 12.948 billion RMB (Wang et al., 2018). Therefore, there is an urgent need to develop new therapeutic strategies for kOA.

Mesenchymal stromal cells (MSCs)-based cell therapy has attracted increasing attention and is developing rapidly in recent years. MSCs are adult multipotent stromal cells of mesodermal origin, which reside in diverse tissues, such as bone marrow, adipose tissue, and placenta (Hou et al., 2016; Zhang et al., 2017). A number of studies have shown that MSCs with anti-inflammatory and immune regulation activities can differentiate into cartilage, bone, fat, tendon and other mesenchymal cell lines (Ham et al., 2015; Mora et al., 2018; Fuggle et al., 2020). Therefore, in field of orthopaedics, MSCs are considered to have anti-kOA potential for cartilage regeneration and repair. Placenta-derived MSCs (PMSCs) are derived from clinically abandoned placenta tissue, which have high proliferation and pluripotent differentiation capabilities (Poloni et al., 2012; Park et al., 2013). Previous studies showed that PMSCs have lower potential of adipogenesis and higher potential of chondrogenesis than other kinds of MSCs (Fan et al., 2012). In addition, the safety of PMSCs in treating patients have been preliminarily verified in clinical practice (Khalifeh Soltani et al., 2019). These findings provide a great potential of PMSCs in treating kOA. To date, little preclinical evidence on PMSCs’ anti-kOA efficacy was obtained, which requires further investigations.

The above studies mainly focus on the ability of PMSCs to differentiate towards the chondrogenic lineage and to regenerate cartilage. Another group of studies has reported the importance of PMSCs’ paracrine properties in regenerative applications. For instance, PMSCs secrete VEGF, bFGF, and angiogenin to enhance the endothelial angiogenesis by up-regulating angiogenic markers (Tie2 and Ang2) (Chen et al., 2015; Liang T. et al., 2017; Mathew et al., 2020). Besides, PMSCs are capable of suppressing pro-inflammatory environment through inhibiting paracrine actions of inflammatory factors (IFN-γ and IL-17) and upregulating anti-inflammatory factors (TGF-β and IL-10) (Zhu et al., 2020). Inflammatory factors are key mediators of the pathogenesis of kOA, in which IL-17 breaks anabolism/catabolism of cartilage and IFN-γ drives neuropathic pain (Naal et al., 2009; Schaible, 2012; Nees et al., 2019). These findings suggest that PMSCs may improve anabolism/catabolism homeostasis through paracrine action of anti-inflammation.

In this study, a classic rat kOA model was established by anterior cruciate ligament transection (ACLT) to comprehensively evaluate the anti-kOA efficacy of PMSCs by assessing pain behavior, cartilage histopathology and immunohistochemistry. Paracrine is a common action mode of MSCs, since the secretions of MSCs possess regenerative capacities. Thus, this study applied PMSCs-conditioned medium (PMSCs-CM) to evaluate the paracrine action of PMSCs on chondrocytes. This is the first study exploring the anti-kOA efficacy and paracrine action of PMSCs, providing preclinical evidence of PMSCs for kOA therapy.

## Materials and methods

### Reagents

Dulbecco′s Modified Essential Medium/Ham′s F12 (1:1) medium (DMEM/F12) was purchased from Thermo Fisher Scientific (MA, United States ). Iscove′s modified Dulbecco’s medium (IMDM), trypsin (0.25%), 4′,6-Diamidino-2-Phenylindole (DAPI) and Bicinchoninic acid (BCA) were purchased from Thermo Fisher Scientific (MA, United States ). Fetal bovine serum (FBS) was purchased from CellMax (Beijing, China). Phosphate Buffered Saline (PBS), Phosphate Buffered Saline-Tween (PBST) and Low elxctrolyte bovine serum albumin (BSA) were purchased from Sangon Biotech (Shanghai, China). Cell culture plates were purchased from Eppendorf (Hamburg, Germany). Transwell chambers were purchased from Corning (NY, United States ). All-in-One cDNA Synthesis SuperMix kit was purchased from Biotool (TX, United States ). 2 × SYBR Green qPCR Master Mix (low ROX) kit was obtained from Bimake (TX, United States ). Phosphatase inhibitor cocktail was purchased from Bimake (TX, United States ). Nitrocellulose membrane was purchased from Sartorius Stedim (Göttingen, Germany). All primary antibodies were purchased from Cell Signaling Technology Inc. (MA, United States ).

### Human PMSCs preparation and identification

Placenta tissues of normal pregnant women were collected immediately after delivery, and informed consent was obtained from the voluntary puerperae before the collection. The primary PMSCs were isolated from human placenta disc and provided by Boyalife Stem Cell Co., Ltd. PMSCs were cultured as described previously (Fan et al., 2012). The cells were cultured in DMEM/F12 supplemented with 10% FBS. For stromal cell identification, the cells (10^6^ cells/ml) were incubated with antibodies of CD73-PE, CD90-PE, and CD34-PE. The labeled cells were analyzed *via* flow cytometer (BD Accuri C6, NJ, United States ). The third passages of cells were used for both *in vivo* and *in vitro* experiments.

The adipogenic, chondrogenic and osteogenic differentiations of PMSCs were tested by specific staining. According to the manufacturer′s instructions, the cells were respectively incubated in adipogenic, chondrogenic, and osteogenic induction medium for 2 weeks. The medium was replaced every 3 days. After the confirmation of morphological manifestations of differentiation, the cells were fixed with 4% polyformaldehyde for 30 min, followed by washing twice with PBS. Oil Red O, Alcian blue, Alizarin red stainings were applied to determine the adipogenic, chondrogenic and osteogenic differentiations, respectively. An inverted phase microscope was used for observation (Carl Zeiss, Göttingen, Germany).

### Animals

All animal experiments were carried out in accordance with the guidelines of the Committee for the Purpose of Control and Supervision of Experiments on Animals (CPCSEA) and approved by the Ethics Committee of Zhejiang Chinese Medical University (Ethical number: 20190506-14). Shanghai Super B&K Laboratory Animal Co. Ltd. (Certificate number: SCXK (Shanghai) 2018-0006) provided the male Sprague Dawley (SD) rats (Grade SPF II) with body weight of 200 ± 20 g. All rats were housed in standard polypropylene cages and maintained at 22 ± 1°C with a 12:12 light/dark cycle and humidity of 50–60% with free food and water. All rats were acclimatized by housing in the above condition for 1 week before the formal experiments.

### kOA induction and treatment

Rat kOA model was established by employing anterior cruciate ligament transection (ACLT) surgery. As described previously (Kamekura et al., 2005), after anesthesia, the left and right knee joints of rat were incised along the medial edge of the patellar ligament to open the joint cavity, and ACLT was conducted with micro-scissors using surgical loupes. To check the success of ACLT, the anterior drawer test was performed. Afterwards, the joint cavity was washed with sterile saline, and then the incision was closed by suturing the cavity and skin. Antibiotics was used for 3 days after the operation to prevent infection.

After modeling for 8 weeks, the rats were randomly divided into three groups as follows (*n* = 10): NC as normal control group underwent the surgical procedure without ACLT, Model as kOA model group, and PMSCs as PMSCs treated model group. The PMSCs treatment was conducted by intra-articular injection of 50 μL PBS solution containing PMSCs (10^6^ cells/ml) at the same site (the medial edge of the patellar ligament) of joint cavity once a week for 4 weeks. Both the NC and kOA model groups were injected PBS with the same volume and frequency as the PMSCs group.

Moreover, another study compared the effect between PMSCs and PMSCs-CM by applying rat kOA model. The rats were randomly divided into four groups as follows (n = 8): NC as normal control group, Model as kOA model group, PMSCs-CM as PMSCs-CM treated model group, and PMSCs as PMSCs treated model group. The PMSCs group received intra-articular injection of 50 μL PBS solution containing PMSCs (10^6^ cells/ml) at the medial edge of the patellar ligament of joint cavity once a week for 4 weeks. The PMSCs-CM group received PMSCs-CM from the same dose of PMSCs (10^6^ cells/ml) at the same site as the PMSCs group.

### Evaluation of pain behavior parameters

At 1 week after the final treatment, von Frey filaments (range from 0.6 to 26 g, Ugo Basile, Lombardy, Italy) and Plantar Test apparatus (Ugo Basile, Lombardy, Italy) were used to measure the mechanical withdrawal threshold (MWT) and thermal withdrawal latency (TWL) respectively. Briefly, rats were individually placed into the mechanical and thermal testing chambers and given 30 min for acclimatization to the testing environment. The room temperature and humidity were kept stable throughout the experimental period. For the assessment of MWT, von Frey filaments were pressed vertically on the mid-plantar surface of the hind paws of each rat for at least 3 times, holding it for at least 2 s. A positive response for each test was defined as the sharp withdrawal of the hind paws, and the maximal bending forces (g) of the filaments were recorded. For the assessment of TWL, a pain threshold detector was used to thermally stimulate the hind paws with light, a focused beam of radiant heat was positioned beneath the plantar surface of the hind paws for at least 3 times and held for maximal 20 s. TWL was defined as the shortest lighting duration. All measurements were conducted in thrice at 4 min interval to obtain the mean value.

### Histopathological and immunohistochemical analyses

The whole knee joints (including femur, tibia, meniscus, ligaments, and capsule) were dissected, fixed with 4% paraformaldehyde (PFA) in PBS (pH 7.4) for 72 h at 4°C, and decalcified with 10% ethylene-diamine tetra acetic acid (EDTA) (pH 7.4) for 8 weeks at 4°C. After gradient ethanol dehydration and paraffin embedding, each sample was sectioned into 3 μm to obtain complete sagittal plane of the tibial plateau cartilage of the knee joint. Then the wax slices were obtained from the microtome and immersed in water bath at 40°C for spreading. The slices were picked and put on a slide in an oven at 37°C to dry for 12 h. Then the sections with intact tissue structure were selected for histological and immunohistochemical staining. The histological changes of articular cartilage among different groups were evaluated by doubleblind observation, according to Osteoarthritis Research Society International (OARSI) score as described before (Glasson et al., 2010).

Immunohistochemistry (IHC) analysis was conducted to detect the expression of collagenase type II (Col2) and matrix metallopeptidase 13 (MMP13). Replicates of sample sections were incubated at 60°C for 4 h with 0.01 mol/L citrate buffer (pH 6.0, Solarbio, Beijing, China) as antigen retrieval, and then the sections were incubated overnight at 4°C with the primary antibody of MMP13 (mouse anti-MMP13 monoclonal antibody) and Col2 (rabbit anti-Col2 monoclonal antibody), followed by incubation with secondary antibody (PV-9001 for Col2, and PV-9002 for MMP13) (ZSGQ-BIO, Beijing, China) at room temperature for 20 min. After washing with PBS for 3 times, the sections were followed by diaminobenzidine solution (Invitrogen, MD, United States ) for colorimetric visualization. The IHC of Col2 and MMP13 was semiquantified by Image-Pro Plus 6.0 software (Media Cybernetics, MD, United States ) under a light microscope (NIKON 80i, Tokyo, Japan). The data were presented as the pixel percentage of MMP13-positive cells to total cells and Col2-positive area to total area in five random fields.

### Preparation of primary chondrocytes

The primary chondrocytes were isolated from the articular cartilage of allogeneic male SD rats. The fresh cartilage tissues from rat donors were harvested and sliced into small pieces, then the pieces were dispersed in 0.25% trypsin for 40 min at 37°C and then treated with 0.1% collagenase II at 37°C for 4 h. The detached chondrocytes were collected and cultured in IMDM medium containing 10% FBS. The chondrocytes at passage three were used for *in vitro* assays.

### Preparation of conditioned medium of PMSCs and chondrocytes

Conditioned medium (CM) of PMSCs was prepared as described previously (Tooi et al., 2016). Briefly, PMSCs and chondrocytes were cultured in DMEM/F12 and IMDM containing 10% FBS, respectively. When the cell confluence attained about 80%, the medium of each cell line was replaced by serum-free IMDM after extensive washing with PBS. After incubation at 37°C and 5% CO_2_ for 48 h, the medium was collected as CM and centrifuged at 1,500 rpm for 10 min to remove cell debris. PMSCs-CM and chondrocytes-CM were aseptically filtered by 0.22-μm filter and stored at -80°C for further use.

### Cellular experiments

Two experimental systems, including CM-used system and co-culture nested system, were employed to investigate the paracrine effects of PMSCs on chondrocytes. In the CM-used system, chondrocytes were divided into three groups as follows: control group, IL-1β group, and PMSCs-CM group. IL-1β group and PMSCs-CM group were modeled by pre-treatment of IL-1β (10 ng/ml) for 24 h. The control group and IL-1β group were treated with chondrocytes-CM in serum-free IMDM, and the PMSCs-CM group was treated with PMSCs-CM for another 24 h. In the co-culture nested system, chondrocytes were cultured in the lower chamber of 6-well plates and divided into three groups as follows: control group, IL-1β group, and PMSCs group. The IL-1β group and PMSCs group were modeled by pre-treatment of IL-1β (10 ng/ml) for 24 h, and the PMSCs group contained PMSCs cells in upper chamber (3.0 μm pore size; Corning) of 6-well plates for another 24 h.

### Wound healing assay

Chondrocytes in the logarithmic growth phase were inoculated in 6-well plates at a density of 3×10^5^ cells per well and divided to three groups applying the CM-used system. The monolayer was scratched using a sterile 200 μL pipette tip and washed with PBS to remove detached cells. Subsequently, the NC and Model group were treated with 3 ml chondrocytes-CM per well, and the PMSCs group was treated with 3 ml PMSCs-CM per well for 0, 24, and 48 h. The cells were observed and imaged under an inverted microscope (CarlZeiss, Göttingen, Germany). The wound area was calculated by ImageJ 1.47 software as follows: blank area ratio (%) = Ar/Ao × 100%, where Ao represented the area of original wound area, and Ar represented the remaining area of wound at each time point.

### Cell immunofluorescence

Chondrocytes were seeded onto coverslips and maintained in IMDM medium or IMDM medium plus IL-1β (10 ng/ml) in 24-well plates for 24 h, and divided to three groups (NC, Model, and PMSCs groups) applying CM-used system. The NC and Model group were treated with 1 ml chondrocytes-CM contained in IMDM, while the PMSCs group was treated with 1 ml PMSCs-CM contained in IMDM. The culture medium was discarded, and the coverslips were rinsed twice with PBS. The cells were fixed with 4% paraformaldehyde, washed three times using PBS, permeabilized with 0.1% Triton X-100 in PBS for 30 min at room temperature, and washed with PBS three times. Thereafter, the cells were incubated in PBST with 10% BSA for 60 min to block nonspecific antibody binding, incubated with primary PCNA antibody in 1% BSA at 4°C overnight, washed with PBS three times, and incubated with a secondary antibody in 1% BSA for 60 min at 4°C. The nuclei were stained using 10 ng/ml DAPI.

### Quantitative real-time polymerase chain reaction (qRT-PCR)

The relative mRNA expression of targeted genes in chondrocytes was analyzed by qRT-PCR assay on an ABI QuantStudioTM seven Flex Real-Time PCR System (Applied Biosystems; Thermo Fisher Scientific, Inc.). Based on the manufacturer’s instructions, total RNA of chondrocytes in each group were isolated using a TRIzol reagent. 1,000 ng of total RNA in each group was reversely transcribed to synthesize cDNA with the Synthesis SuperMix kit. Then, the SYBR^®^ Premix Ex Taq II (Tli RnaseH Plus), forward primer, and reverse primer of target gene were used in the process of cDNA amplification. The qRT-PCR reaction conditions included pre-incubation at 95°C for 5 min, followed by 40 cycles of denaturation at 95°C for 10 s, annealing and extension at 60°C for 30 s. *β*-Actin was used as a housekeeping gene. The relative mRNA expression level of each target gene was calculated by using the 2^−ΔΔCT^ method. The sequences of forward and reverse primer were presented in Table 1.

**TABLE 1:** Primer sequences of rat target genes.

Gene	Forward primer	Reverse primer
*β-actin*	5′-CCC​GCG​AGT​ACA​ACC​TTC​T-3′	5′-CCC​GCG​AGT​ACA​ACC​TTC​T-3′
*Col2*	5′-CTC​AAG​TCG​CTG​AAC​AAC​CA-3′	5′-GTC​TCC​GCT​CTT​CCA​CTC​TG-3′
*Sox9*	5′-CAT​CAA​GAC​GGA​GCA​ACT​GA-3′	5′-TGT​AGT​GCG​GAA​GGT​TGA​AG-3′
*Adamts4*	5′-TTC​GCT​GAG​TAG​ATT​CGT​GGA​G-3′	5′-CGG​ACT​TTT​ATG​TGG​GTT​GC-3′
*Adamts5*	5′-TGG​AGT​GTG​TGG​AGG​GGA​TA-3′	5′-CGG​ACT​TTT​ATG​TGG​GTT​GC-3′
*Mmp13*	5′-CTA​TGG​TCC​AGG​AGA​TGA​AGA​C-3′	5′-GTG​CAG​ACG​CCA​GAA​GAA​TCT-3′

### Western blot analysis

Chondrocytes were washed with PBS for 3 times and lysed with RIPA lysis buffer (50 mM Tris-HCl, pH 7.4, 150 mM NaCl, 1 mM EDTA, 1% Triton and 0.1% SDS) supplemented with protease inhibitor cocktail and phosphatase inhibitor cocktail. The mixture was homogenized and lysed on ice for 30 min. The protein concentration was determined by the BCA method. The same amount of protein sample was separated by denaturing sodium dodecyl sulfate polyacrylamide gel electrophoresis (SDS-PAGE; 6–12%) and transferred to a nitrocellulose membrane. The membrane was blocked with 5% skim milk for 2 h at 4°C and incubated at 4°C overnight with the primary antibody against β-actin, β-tubulin, Col2, MMP13, and Sox9. After washing with TBST for 3 times, the membrane was incubated with peroxidase-conjugated goat antirabbit/mouse IgG at room temperature for 1 h, each protein was visualized using Western Lightning^®^ Plus ECL (Perkin Elmer, Inc., Waltham, MA, United States ), detected using X-ray film (Kodak, Tokyo, Japan) and scanned.

### Statistical analysis

Data were expressed as mean ± standard deviation (SD). One-way analysis of variance (Pers et al.) with Least Significant Difference (LSD) post hoc multiple comparison test was applied to evaluate the statistically significant difference between groups. All statistical analyses were performed using the IBM SPSS software (SPSS Statistics V22, IBM Corporation, United States ). *p* values < 0.05 were considered statistically significant.

## Results

### Characterization of PMSCs

**FIGURE 1:**
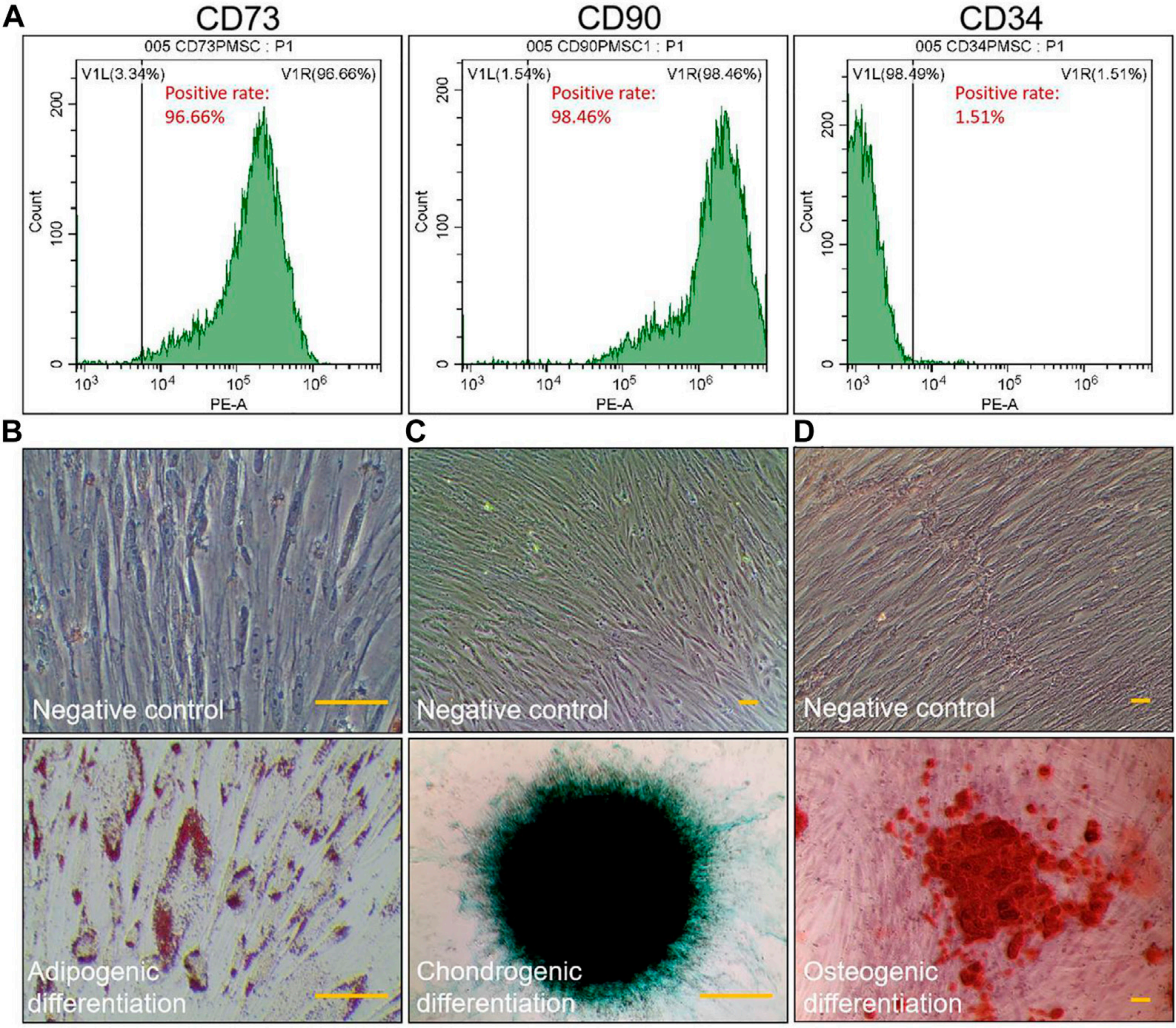
Identification of PMSCs. Immunophenotype of PMSCs surface markers determined by flow cytometry **(A)**. Specific staining of the cells after inducing three-line differentiation (scale bar = 25 μm): adipogenic differentiation was examined by Oil Red O staining **(B)**, chondrogenic differentiation was examined by Alcian blue staining **(C)**, and osteogenic differentiation was examined by Alizarin red staining **(D)**. Negative control: images of PMSCs cultured in non-induction medium for 2 weeks. All experiments were repeated for three times.

The results of flow cytometry indicated that the surface markers’ positive rates of CD73, CD90, and CD34 of PMSCs were 96.66%, 98.46% and 1.51%, respectively, indicating a similar surface marker profile as mesenchymal stromal cells (CD73 > 95%, CD90 > 95%, and CD34 < 2%) (Figure 1A). After the differentiation induction, the cells showed specific phenotypes of adipogenesis, chondrogenesis, and osteogenesis (Figures 1B–D).

### Therapeutic efficacy of PMSCs on kOA rats

**FIGURE 2:**
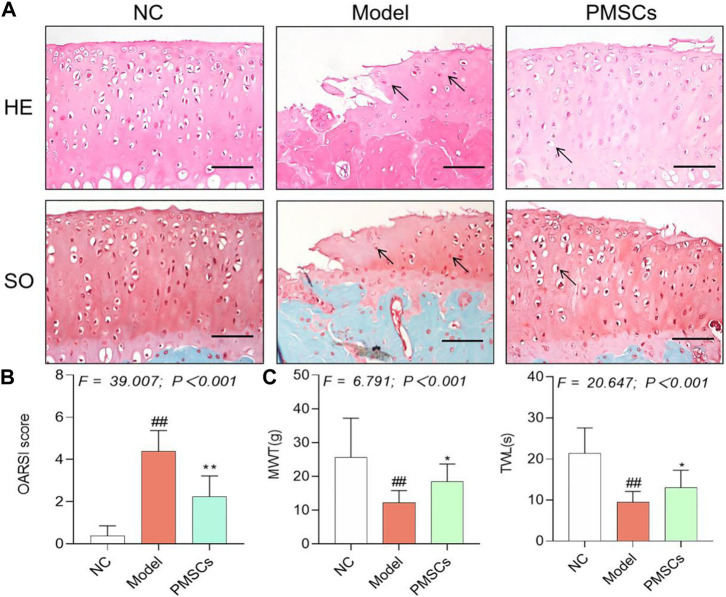
Histopathological and pain behavioral evaluations of the *in vivo* effects of PMSCs on kOA rats. **(A)** Safranin O/Fast green staining and H&E staining with black arrows (hypertrophy or loss of chondrocytes). Scale bars = 50 μm; **(B)** OARSI scoring of histopathology; **(C)** Measurement of MWT and TWL of rats. Values were presented as mean ± SD. ^*##*^ *p* < 0.01 vs. NC group; *p < 0.05 vs. Model group; **p < 0.01 vs. Model group. 10 rats from each group were used per each analysis and all experiments were repeated at least three times.

**FIGURE 3:**
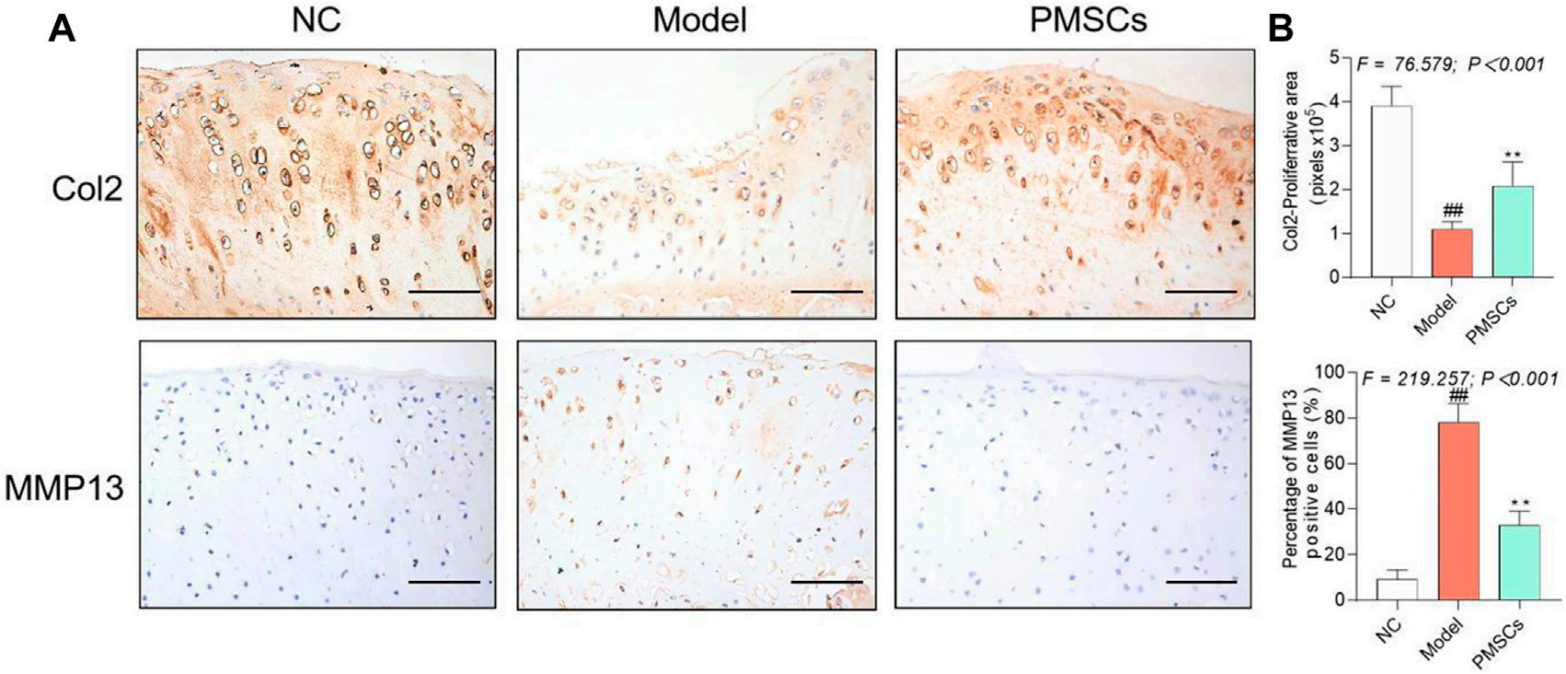
IHC evaluation of the *in vivo* effect of PMSCs on anabolism/catabolism. **(A)** IHC staining of Col2 and MMP13 on cartilage; Scale bars = 50 μm. **(B)** Quantitative measurement of positive area percentage of Col2 and positive cell percentages of MMP13. Values were presented as mean ± SD. ^*##*^ *p* < 0.01 vs. NC group; *p < 0.05 and **p < 0.01 vs. Model group. 10 rats from each group were used per each analysis and all experiments were repeated at least three times.

Pain behavior, histopathological observation, and immunohistochemical analyses were applied to evaluate the *in vivo* effects of PMSCs*.* As shown in Figures 2A,B, severe kOA-like degeneration of cartilage, such as chondrocyte loss and collagen matrix defect, were developed in the Model group at 8 weeks after the ACLT surgery, with significant increased OARSI score (*p* < 0.01 vs. NC). By contrast, intra-articular injection of PMSCs in the PMSCs group significantly restored the cartilage against degeneration by increasing chondrocytes and improving structural integrity of cartilage, with significantly decreased OARSI score (*p* < 0.01 vs. Model), indicating chondroprotective effect of PMSCs. As shown in Figure 2C, the pain parameters, TWL and MWT, were significantly abnormal in the Model group (each *p* < 0.01 vs. NC) and were restored in the PMSCs group (each *p* < 0.05 vs. Model). Moreover, as shown in Figure 3, the Col2 and MMP13 expressions of cartilage in the Model group were significantly downregulated and upregulated, respectively (each *p* < 0.01 vs. NC), the abnormalities of which were significantly restored in the PMSCs group (*p* < 0.01 vs. Model), indicating improving effect of PMSCs on the cartilage anabolism/catabolism.

### Molecular actions of PMSCs on IL-1β-modeled chondrocytes

**FIGURE 4:**
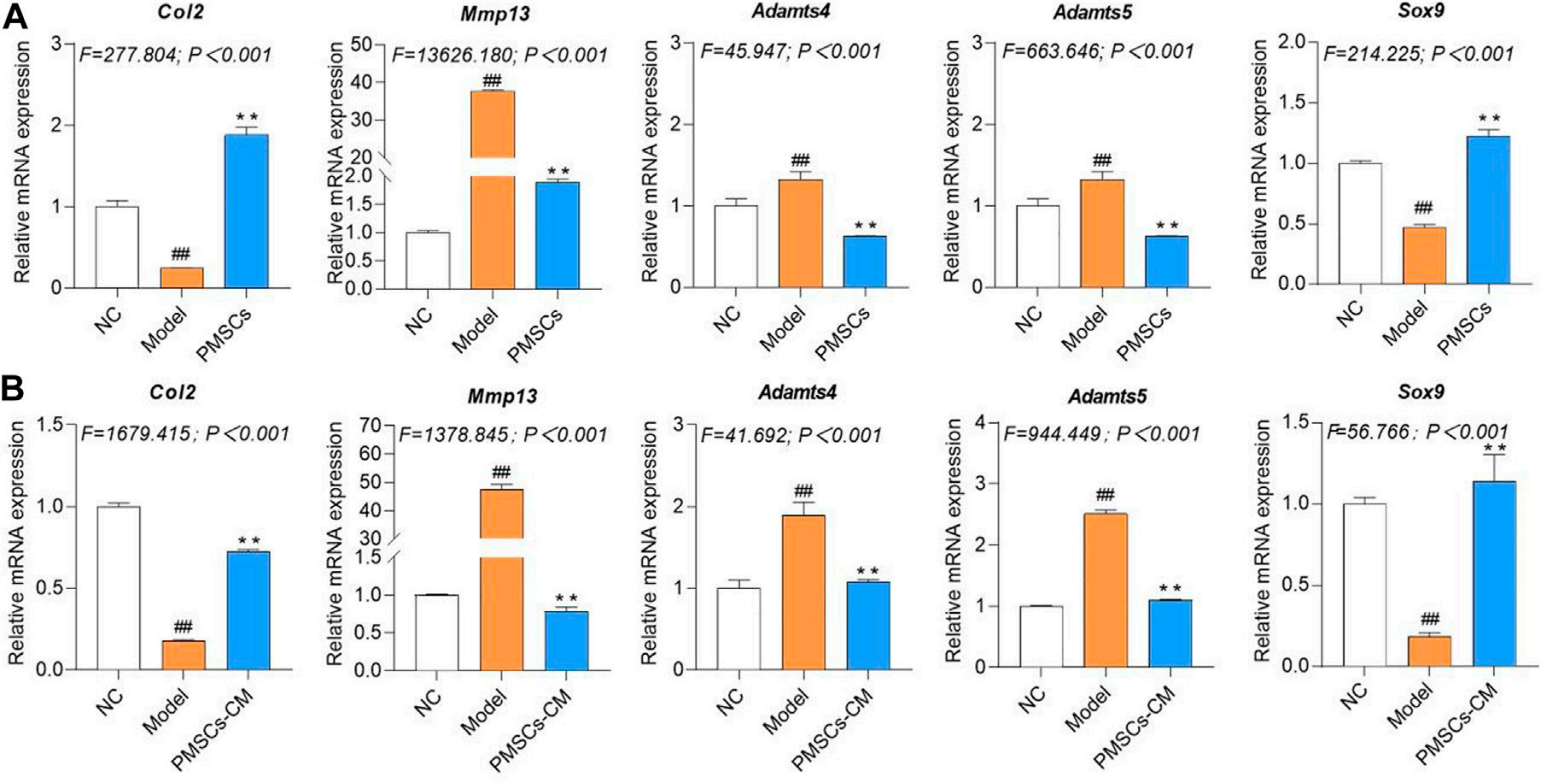
Regulations of PMSCs and PMSCs-CM on IL-1β-treated chondrocytes. **(A)** mRNA expressions of chondrocytes in co-culture with PMSCs; **(B)** mRNA expressions of chondrocytes treated with PMSCs-CM. Values were presented as mean ± SD. ^*##*^ *p* < 0.01 vs. NC group; **p < 0.01 vs. Model group. All experiments were repeated at least three times.

**FIGURE 5:**
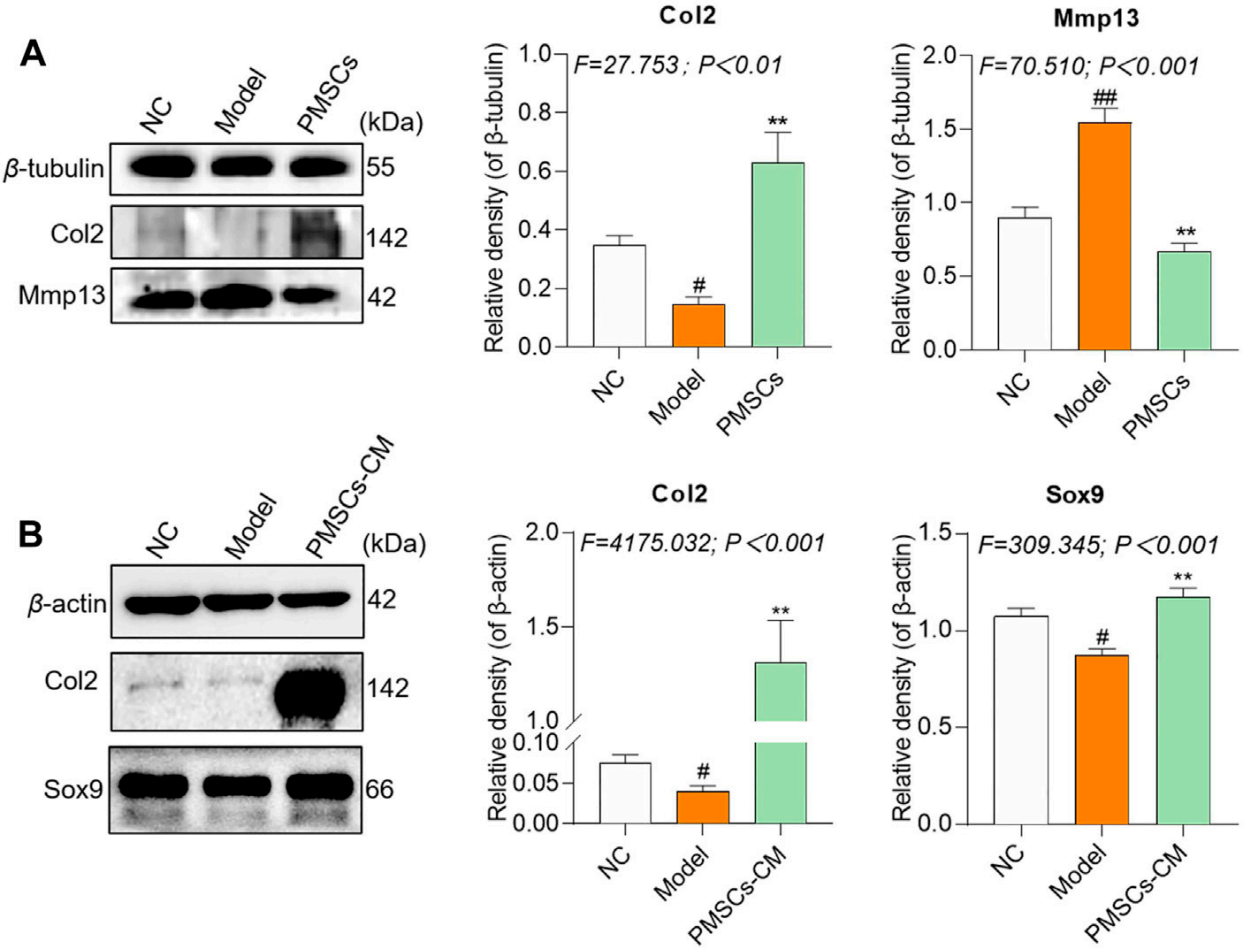
Regulations of PMSCs and PMSCs-CM on IL-1β-treated chondrocytes. **(A)** Expressions of target proteins in rat chondrocytes treated with PMSCs. **(B)** Expressions of target proteins in rat chondrocytes treated with PMSCs-CM. Values were presented as mean ± SD. ^*#*^ *p* < 0.05 vs. NC group; ^#*#*^ *p* < 0.01 vs. NC group; *p < 0.05 and **p < 0.01 vs. Model group. All experiments were repeated at least three times.

To clarify the molecular actions of PMSCs on IL-1β-modeled chondrocytes, nested co-culture using PMSCs as well as direct culture using PMSCs-CM were applied for qRT-PCR analysis. As shown in Figure 4A, in the co-culture system, the chondrocyte mRNA expressions of anabolic genes (*Col2* and *Sox9*) were significantly downregulated and that of catabolic genes (*Mmp13*, *Adamts4* and *Adamts5*) were upregulated in the Model group (each *p* < 0.01 vs. NC), while these abnormalities were restored to the NC levels in the PMSCs group (each *p* < 0.01 vs. Model). As shown in Figure 4B, a similar tendency was seen in the direct culture system, in which the anabolic gene (*Col2* and *Sox9*) and catabolic genes (*Mmp13*, *Adamts4* and *Adamts5*) were abnormally expressed in the Model group (each *p* < 0.01 vs. NC) and restored in the PMSCs-CM group (each *p* < 0.01 vs. Model). As shown in Figures 5A,B, the WB data further confirmed that the anabolic (Col2 and Sox9) and catabolic (Mmp13) molecules of chondrocytes were abnormally expressed in the Model group (each *p* < 0.05 vs. NC) and were restored in the PMSCs (co-culture) group or in the PMSCs-CM group (each *p* < 0.01 vs. Model). The above data demonstrated that PMSCs and PMSCs-CM exerted similar regulatory effects on the anabolism/catabolism of IL-1β-modeled chondrocytes.

### Paracrine effects of PMSCs on IL-1β-modeled chondrocytes

**FIGURE 6:**
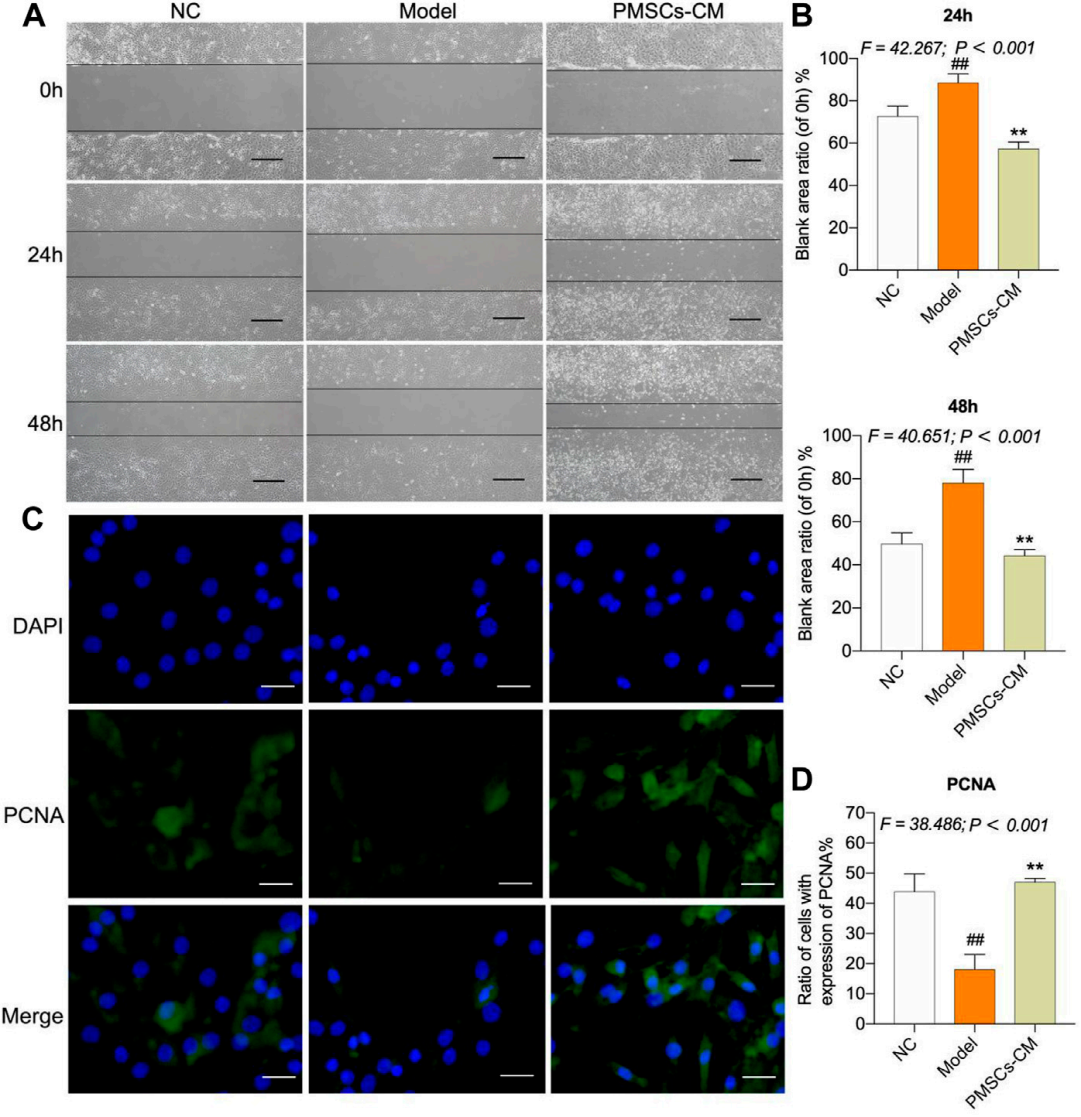
Effects of PMSCs-CM on the wound healing and PCNA expression of chondrocytes. **(A)** Wound healing assay of chondrocytes with PMSCs-CM treatment at 0 h, 24 h and 48 h. Scale bars = 200 μm; **(B)** Quantification of blank area ratio from 24 h to 0 h and from 48 h to 0 h; **(C)** Cell immunofluorescence assay of chondrocytes at 24 h after PMSCs-CM treatment; **(D)** Quantification of cell ratio (PCNA-expressed cell number/total cell number). Values were presented as mean ± SD. ^*##*^ *p* < 0.01 vs. NC group; **p < 0.01 vs. Model group. All experiments were repeated at least three times.

Since the wound healing and proliferation ability of chondrocytes may play a crucial role in the regeneration and chondroprotection of cartilage damage in kOA, we conducted wound healing assay and cell immunofluorescence assay to evaluate the chondroprotective effects of PMSCs on IL-1β-modeled chondrocytes. PMSCs-CM was used in these assays to reveal the paracrine action of PMSCs. As shown in Figures 6A,B, the ratios of wound area from 24 h to 0 h and from 48 h to 0 h in the Model group were significantly higher than the NC levels (each *p* < 0.01 vs. NC), while that in the PMSCs-CM group were significantly lower than the Model levels (each *p* < 0.01 vs. Model). Moreover, the ratios in the PMSCs-CM group were even lower than the NC levels, indicating a dramatic effect of PMSCs-CM on the wound healing of chondrocytes. As shown in Figures 6C,D, the PCNA expression of chondrocytes was significantly inhibited in the Model group (*p* < 0.01 vs. NC), and in contrast the inhibition was restored to the normal level in the PMSCs-CM group (*p* < 0.01 vs. Model; (*p* > 0.05 vs. NC).

## Discussion

Although a large number of clinical and preclinical studies have confirmed that MSCs from various origins can improve kOA (Zhang X. T. et al., 2021; Chen et al., 2021; Feng et al., 2021; Jin et al., 2021; Noor et al., 2021), few of them focused on the efficacy of PMSCs on kOA at present (Khalifeh Soltani et al., 2019). To complement this knowledge, we applied an ACLT rat kOA model to evaluate the therapeutic efficacy of PMSCs and applied IL-1β-modeled chondrocytes to evaluate the paracrine actions of PMSCs. Inflammation is a key driver in the pathogenesis of kOA, characterized by increased levels of proinflammatory cytokines (e.g., IL-1β), which, in turn, induces ECM degradation through enhanced production of catabolic enzymes (Wehling et al., 2009; Goldring and Otero, 2011; Kapoor et al., 2011; Rigoglou and Papavassiliou, 2013). Therefore, many studies have applied IL-1β to establish *in vitro* model of kOA on chondrocytes, since IL-1β induces inflammatory damage on chondrocytes and impels chondrocytes to catabolic MMP1 and MMP13 to destruct cartilage matrix (Guan et al., 2018; Zhang et al., 2020; Chen et al., 2021; Ohashi et al., 2021; Pferdehirt et al., 2022). In this study, IL-1β was used to treat chondrocytes, resulting in increased chondrocyte degradation (Adamts4, Adamts5, MMP13) and decreased synthesis (Col2, Sox9), indicates the kOA-like phenotype of chondrocytes.

The *in vivo* data demonstrated that PMSCs attenuated the progression of kOA by alleviating joint pain and protecting cartilage against degeneration (Figures 2, 3), but whether this effect was a direct analgesic effect or a subsequent effect after chondroregeneration was unclarified. Further studies are needed to determine this point. The reason why we injected PMSCs into both knees of rats as the treatment is that: 1) the experimental samples (joints) can be doubled so that we can have more tests; 2) one side treatment would not show enough effect on pain behavior since the other side still produces pain; and 3) this design of experiment can mimic the cases in which many patients have kOA on both knees. The *in vitro* data demonstrated that PMSCs exerted paracrine chondroprotective effects by improving anabolism and inhibiting catabolism (Figures 4, 5). The innovative points of this study were the determination of the anti-kOA efficacy of PMSCs and its paracrine actions. The comparison between the PMSCs-CM and PMSCs was conducted *in vitro* and *in vivo*, and the results showed that PMSCs-CM exerted similar benefits of chondroprotection as PMSCs themselves (Figures 4, 5, Supplementary Figures S1, 2). The reason for the use of different medium is to remove the DMEM-F12 in CM so as to avoid the influence of DMEM-F12. By using the CM in serum-free IMDM, the real effect of CM can be observed. Sox9 and Col2 are the representative anabolic parameters that have been activated by PMSCs. Sox9 initiates chondrogenesis and regulates cell metabolism by inhibiting fatty acid oxidation, thereby improving regeneration of cartilage (van Gastel et al., 2020). Col2, as the main component of cartilage matrix, maintains structural integrity and function of cartilage (Henrotin et al., 2007; Antunes et al., 2020). MMP13, Adamts4 and Adamts5 are the representative catabolic parameters that have been inhibited by PMSCs. MMP13 degrades not only type II collagen, but also proteoglycan, type IV and IX collagen, and osteonectin in cartilage, which participates in the cartilage degeneration in kOA (Zhang et al., 2019; Phillips, 2021). Adamts4 and Adamts5 mediate the degradation of extracellular chondrogenic proteins, leading to the loss of cartilage integrity and accelerating the progression of kOA (Verma and Dalal, 2011; Malemud, 2019). Moreover, PMSCs-CM contains many components, including exosomes, growth factors, and cytokines, which per sé can affect the expression of collagen, MMPs and other parameters in chondrocytes (Xu et al., 2016; Shin et al., 2021). However, the fact that which component of PMSCs-CM determined the affection remains unknown and warrants further investigations.

Many of the published studies have investigated the anti-kOA effects of other sources-derived MSCs by applying the same *in vivo* model or *in vitro* model as ours. Human adipose-derived MSCs (hADSCs) and human umbilical cord-derived MSCs (hucMSCs) have been investigated by using the rat model of kOA induced by ACLT. The results showed that hADSCs and hucMSCs exerted chondrogenic potential and significantly attenuated the development of kOA in rats (Ju et al., 2022). The effects of hADSCs and hucMSCs on the pathological phenotype were similar to that of PMSCs in our study. Human synovial MSCs (hsMSCs) was also applied to treat ACLT-induced kOA model in rats, which protected articular cartilage and reduced the OARSI score (Ozeki et al., 2016). The reduction degree of OARSI score with hsMSCs treatment was smaller than that with PMSCs treatment in this study. In addition, the effect of hsMSCs on cartilage anabolism/catabolism was not shown, while PMSCs improved anabolism and inhibited catabolism to protect cartilage from degradation. Similar *in vitro* studies have shown that chondrocytes were modeled by IL-1β and treated with MSC-CM (Ohashi et al., 2021; Sampath et al., 2021; Lofgren et al., 2022). The decreased expression of MMP13 and Adamts5 and increased expression of Col2 were observed (Sampath et al., 2021), which showed similar tendency of results with ours. Although there have been no report of the anti-kOA treatment of PMSCs, the comparable effects of PMSCs to other sources-derived MSCs indicates that PMSCs is a promising and alternative candidate of allogeneic MSCs for kOA therapy, especially when PMSCs are easily available.

MSCs-based cell therapy has promising potential in treating kOA (Fan et al., 2012; Poloni et al., 2012; Park et al., 2013). Generally, the MSCs commonly used in clinical practice are autologous. For instance, autologous intra-injection of bone marrow stem cells (bMSCs) to kOA patients resulted in amelioration of joint pain and improvement of articular cartilage after 12 months of follow-up (Orozco et al., 2013). Besides, autologous adipose stem cells (ADSCs) also resulted in satisfactory functional improvement and pain relief of kOA patients (Pers et al., 2016; Lee et al., 2019). However, the autologous bMSCs and ADSCs have potential disadvantage of aged cells derived from the aged donors, resulting in risks of impaired capacities and reduced proliferation (Murphy et al., 2002; Chua et al., 2014)*.* In addition, the autologous collection process is an extra damage on patients. In this respect, allogeneic application of stromal cells are superior to the autologous one because young allogeneic cells (e.g., PMSCs from neonatal tissues) are accessible and may have better capacities (Ding et al., 2021). PMSCs are regarded as the ideal allogeneic stromal cells for cell therapy of kOA, owing to their higher chondrogenic potential and lower adipogenic potential than other kinds of MSCs (Khalifeh Soltani et al., 2019). In previous clinical report, intra-articular injection of PMSCs was applied to treat 10 patients with mild kOA and significant relief of kOA symptoms were found followed up for 24 weeks (Khalifeh Soltani et al., 2019). Moreover, PMSCs embedded in silk fibroin scaffold repaired the cartilage defects of rabbits (Li et al., 2012). These findings suggest anti-kOA potential of PMSCs, which was further confirmed by this study. Compared with other MSCs, PMSCs can be derived from well-sourced medical wastes in a non-invasive way, producing little ethical controversy.

There may be some concerns about the allo reactivity against allo PMSCs in the treatment of rat kOA, but a large number of studies have applied allo MSCs without any reported allo reactivity (Natsumeda et al., 2017; Rosa et al., 2022). For instance, endocardial injections of allo MSCs to a porcine model of ischemic cardiomyopathy showed that allo MSCs did not produce any adverse immune response (Natsumeda et al., 2017), and articular injections of allo MSCs into horse joint cavity did not result in immune recognition on the injection (Rosa et al., 2022). Also, in this study, we did not find any allo reactivity in rats after the injections of allo PMSCs, indicating the safety of allo PMSCs with no allo reactivity.

The known paracrine factors of PMSCs have been found to promote endothelial cell migration, angiogenesis, nerve protection, skin wound repair, and myelin sheathes regeneration (Liang L. et al., 2017; Kumar et al., 2019), verifying that paracrine action is the basis of PMSCs-based regenerative therapy. Although we demonstrated the PMSCs’ anti-kOA efficacy and its paracrine action in this study, it was unclear which paracrine factors played the major role and whether there were synergistic effects in the PMSCs secretome. Further studies are warranted for identifying the paracrine factors of PMSCs and exploreing their roles and contributions in kOA treatment.

## Conclusion

This study demonstrated that PMSCs ameliorated joint pain and cartilage degeneration in kOA rats through actions of chondroprotection and chondroregeneration. Our findings provide new supporting evidence of the anti-kOA efficacy of PMSCs and determines a possible paracrine mode of action of PMSCs, suggesting that it may be a promising approach for kOA stromal cell therapy. The paracrine effects of PMSCs *in vivo* need to be further investigated.

## Data Availability

The original contributions presented in the study are included in the article/Supplementary Material further inquiries can be directed to the corresponding authors.
