# Supplementary material for: Intra-articular injection of placental mesenchymal stromal cells ameliorates pain and cartilage anabolism/catabolism in knee osteoarthritis
Source: Front Pharmacol. 2022 Nov 29;13:983850. doi: 10.3389/fphar.2022.983850 (PMC9745038; doi:10.3389/fphar.2022.983850)
Supplement: Supplementary file 1 [file DataSheet3.PDF]

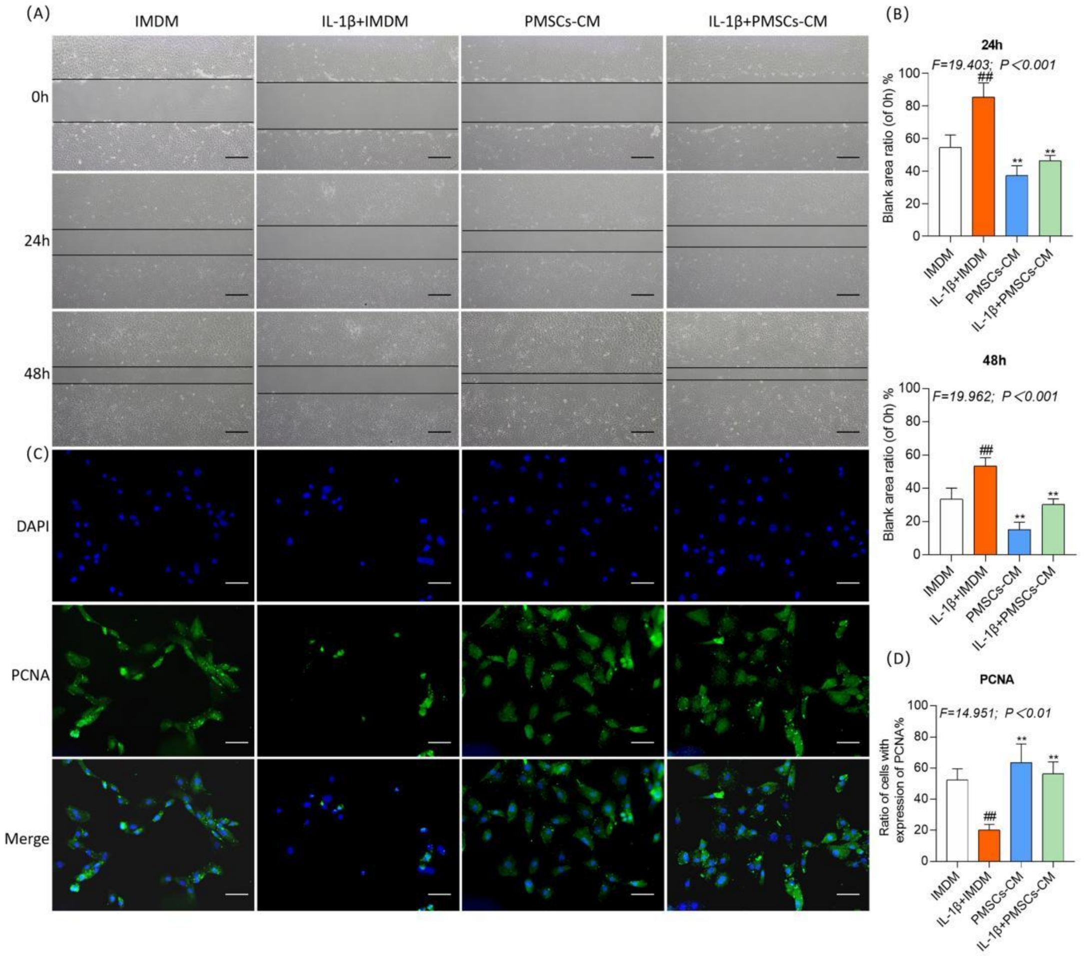

Figure S1. Effects of PMSCs-CM on the wound healing and PCNA expression of chondrocytes. (A) Wound healing assay of chondrocytes with PMSCs-CM treatment at 0 h, 24 h and 48 h. Scale bars = 200 μm; (B) Quantification of blank area ratio from 24 h to 0 h and from 48 h to 0 h; (C) Cell immunofluorescence assay of chondrocytes at 24 h after PMSCs-CM treatment; (D) Quantification of cell ratio (PCNA-expressed cell number/total cell number). Values were presented as mean ± SD. $^{##}P < 0.01$ vs. NC group; $^{**}P<0.01$ vs. Model group. All experiments were repeated at least three times.

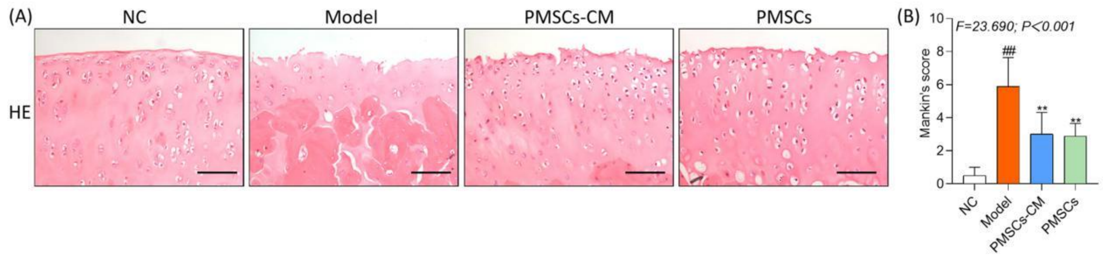

Figure S2. Histopathological evaluations of the *in vivo* effects of PMSCs-CM and PMSCs on rats after modeling (n = 8). (A) H&E staining. Scale bars = 50 μm; (B) Mankin′s scoring of histopathology; Values were presented as mean ± SD. $^{##}P < 0.01$ vs. NC group; $^{*}P < 0.05$ vs. Model group; $^{**}P < 0.01$ vs. Model group. NC and Model group were treated with blank medium (IMDM).
